# Effect of SQW on the bladder function of mice lacking TRPV1

**DOI:** 10.1186/s12906-016-1420-6

**Published:** 2016-11-15

**Authors:** Huanling Lai, QiTao Yan, Hongying Cao, Pengyu Chen, Yifei Xu, Weiwen Jiang, Qinghe Wu, Ping Huang, Bo Tan

**Affiliations:** 1 School of Chinese Materia Medica, Guangzhou University of Chinese Medicine, Guangzhou, 510006 China; 2 Institute of Chinese Medical Sciences, University of Macau, Macau, 999078 China; 3 School of Fundamental Medical Science, Guangzhou University of Chinese Medicine, Guangzhou, 510006 China

**Keywords:** TRPV1, Suo Quan Wan, TRPV1^−/−^ mice, Pelvic nerve firing, Urodynamic, P2X3

## Abstract

**Background:**

Suo Quan Wan (SQW) is an effective traditional Chinese prescription on treated lower urinary tract symptoms (LUTS), and has been proved have modulation effect on the expression of transient receptor potential vanilloid 1 (TRPV1) in accordance with the recovery of bladder function of overactive bladder rat. This study further investigated the mechanism of SQW modulated TRPV1 signaling and bladder function using TRPV1 knockout (KO) mice.

**Methods:**

Study was conducted using wild type and TRPV1 KO mice. The KO animals were grouped into KO group and SQW treated group. We applied in vivo cystometrogram recording techniques to analyze voiding control of the urinary bladder, as well as in vitro organ bath to study bladder distension response to various compounds, which subsequently elicited normal smooth muscle excitation. Real-time polymerase chain reaction and western blot analysis were performed to quantify the expression of TRPV1 and P2X3 in the bladder. ATP released from bladder strips was measured using the luciferin–luciferase ATP bioluminescence assay kit.

**Results:**

KO preparation inhibited decrease micturition times, while micturition interval and volume were increased. Results of urodynamic record of the TRPV1^−/−^ mice during NS infusion showed reduced bladder pressure and contraction which exhibited decreased response to α, β-me ATP, KCl, and carbachol and no response to CAP. The ATP released by the TRPV1^−/−^ mice from strips of bladder smooth muscles was significantly reduced, along with no TRPV1 expression and reduced expression level of P2X3 in the bladder. SQW could increase ATP release in some degree, while had no effect on TRPV1 and P2X3 expression. SQW could improve bladder pressure slightly, while make no significantly effects on the force response to α,β-meATP, CAP, carbachol in gradient concentration, and KCl, as well as MBC and voiding activities.

**Conclusions:**

TRPV1 plays an important role in urinary bladder mechanosensitivity. The effective SQW is hard to play its proper role on bladder function of mice without TRPV1.

**Electronic supplementary material:**

The online version of this article (doi:10.1186/s12906-016-1420-6) contains supplementary material, which is available to authorized users.

## Background

Efficient bladder micturition is triggered primarily by bladder afferent nerve activities and the synthesis of somatic and autonomic efferent mechanisms that coordinate detrusor contraction and sphincter relaxation during bladder distension. The dysfunctions of these normal pathways are probably related to lower urinary tract storage disorders, such as urinary incontinence and overactive bladder symptom syndrome (OAB) [1, 2]. In the field of urology, TRPV1 has been extensively studied for several decades. The role of TRPV1 receptors has also been analyzed in various urinary tract pathologies. As a pressure sensor, TRPV1 mediates stretch detection and regulates sensory function in the bladder [3, 4]. Clinical evidence has proven that modulating the function of TRPV1 signaling is related to treating many lower urinary tract symptoms (LUTS) [5, 6]. Direct or indirect stimulation of afferent nerve fibers modulated afferent information to the central nervous system (CNS), consequently influenced bladder filling or voiding. One of the mechanisms underlie TRPV1 induced increased contraction is the hypothesis that TRPV1 is expressed by the epithelial cells of the transitional epithelium, and that the activation of these TRPV1-expressing cells results in ATP release, which consequently activates P2X3 receptors lead to bladder smooth muscle contraction [7].

Many Chinese traditional prescription have bladder functional regulation effect and have been used in the clinical treatment of LUTS in China, Suo Quan Wan (SQW) [8] and Ba-Wei-Di-Huang-Wan (Hachi-mi-jio-gan) [9] are the representative prescription. SQW, which comprised *A. oxyphylla* Miq, *Dioscorea rhizome* Thunb., and *Aconitii tuber*, is speculated to have a relaxant effect on acetylcholine-induced contraction of smooth muscles and is used clinically in the treatment of LUTS [9]. Previously, we used OAB model rats, in which TRPV1 was highly expressed in the bladder, to investigated the treated effect of SQW on OAB. Results demonstrated that the highly expression level of TRPV1 in the bladder after induction of BOO, decreased after treated with SQW, and showed dose-dependent effects. Moreover, results of TRPV1 expression in the bladder are in agreement with urodynamic change, according to the induction of OAB model and SQW treatment. This studies have provides evidences that treatment of SQW on the bladder function of OAB is related to TRPV1 modulation effect [8].

Based on this previous studies, we put forward a hypothesis that SQW modulating bladder function maybe related with the functional interaction of TRPV1 and P2X3. Thus, we further investigated the mechanisms of SQW on TRPV1 signaling by using TRPV1 knockout (KO) mice. We applied in vivo cystometrogram recording techniques to analyze the urinary bladder function of TRPV1 KO mice, as well as in vitro organ bath to study bladder distension response to various compounds, which subsequently elicited normal smooth muscle excitation. ATP released from bladder strips was measured using the luciferin–luciferase ATP bioluminescence assay kit. The expression of TRPV1 and P2X3 in mice bladder was measured via real-time polymerase chain reaction (RT-PCR) and western blot analysis. Above to further study the mechanism of SQW on TRPV1 regulation.

## Methods

### Animals and experimental grouping

Adult female C57BL/6 wild type (WT, 10–12 weeks, 18–22 g, *n* = 10) and TRPV1 gene KO (TRPV1^−/−^, KO) mice (10–12 weeks, 18–22 g), Jackson Laboratories (*n* = 32), were used in this study. The TRPV1^−/−^ KO mice were grouped into the TRPV1 KO group, the SQW high dosage (1170 mg/kg) treated group (SQW H), and the SQW low dosage (585 mg/kg) treated group (SQW L), the selection of dosage of SQW is according to our previous studies [8]. The animals were kept in a regulated environment, with free access to food and water, and maintained on a 12 h:12 h light/dark cycle. No overt difference in feeding behavior, litter size growth rate, and body weight was observed among the four groups.

### Drug preparation

In our study, the SQW was purchased from Hunan Hansen Pharmaceutical Co. Ltd. Briefly, the process and production are as follows, all these three components are weighed in the ratio of 1:1:1 and well-mixed after grinded into powder. Using appropriate distilled water to help these powder make into pills. According to the Chinese Pharmacopeia [10], assurance of quality control for SQW is validated and linderane is the recorded reference standard of SQW. HPLC and TLC were used to test these typical chemicals of SQW in our present experiment [11] (Data submitted as Additional file 1).

### Voiding stain on paper (VSOP) analysis

**Fig. 1:**
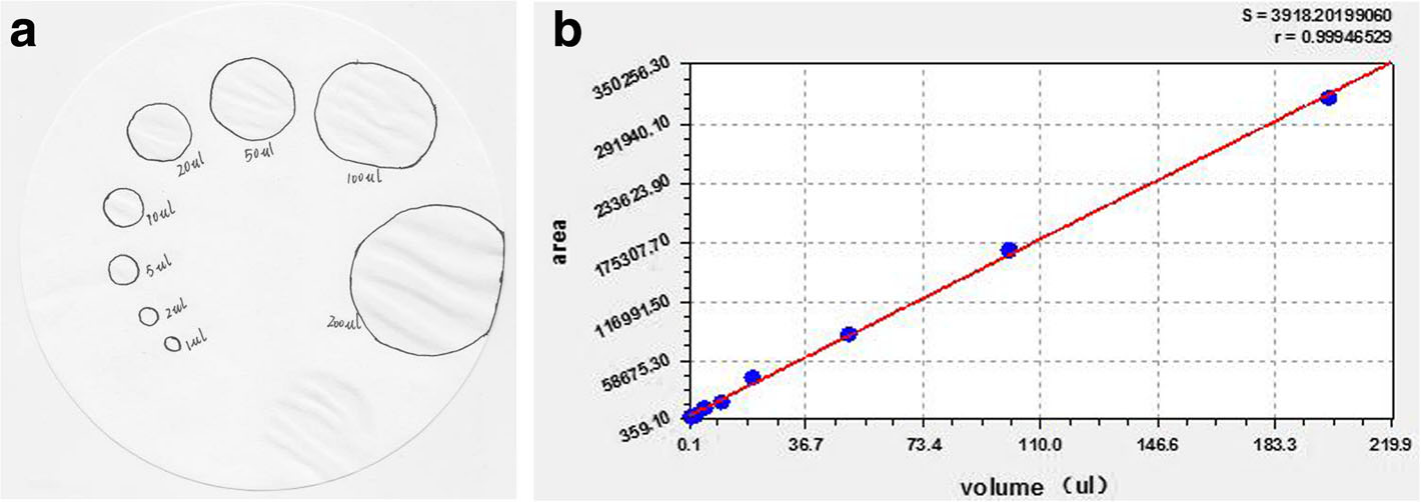
**a** Standard curve of VSOP. The surface area is make by fluid drops of known volume (1 μL, 2 μL, 5 μL, 10 μL, 20 μL, 50 μL, 100 μL, 200 μL). **b** Linear correlation between liquid volume and stained area on the filter paper within the range of 50–800 μL, *r* = 0.9994, y = 4830.30 x + 1586.07 (**b**)

The mice were individually kept in metabolic cages. Food and water were unlimited, and the mice were allowed to adapt to the new environment for 24 h. Urine output was measured by evaluating the surface area of the stains on the paper used for VSOP analysis (Whatman) for 3 h after the mice were placed in the cages. The collected papers were imaged under ultraviolet light to visualize the urine area and analyzed using the edge-detection function of ImageJ software to determine the surface area of the individually voided urine spots. The voiding volumes of each mouse were calculated based on a calibration curve of surface area versus fluid drops of known volume (1 μL, 2 μL, 5 μL, 10 μL, 20 μL, 50 μL, 100 μL, 200 μL) (Fig. 1a).

### Urodynamic test

Urodynamic evaluation was performed using a urodynamic measuring device (Laborite Delphis 94-R01-BT, Canada). Mouse were anesthetized by administration of 10 % urethane (4.0 mg/kg) [12]. The bladder was emptied and a two-barrel Polyethylene tubing with a heat-flared end was inserted into the dome of the bladder and secured by a silk suture. One barrel of the tubing was connected to a pressure transducer for continuous measurement of intravesical pressures, another was connected to a Harvard syringe pump for infuse saline into the bladder. After the bladder was emptied, cystometrography was performed using saline infusion at 50 μL/min. Maximum voiding pressure (MVP) and bladder pressure (BP) were then measured. Pumping was stopped at that moment when urine was observed at the external orifice of the urethra. Maximum bladder capacity (MBC) was calculated as infusion speed multiplied by time [13]. The values for the individual mouse represent the means of two or three voiding cycles.

### In vitro experimental protocols

The mice were anesthetized with urethane (Halocarbon Laboratories, USA), and the urinary bladder was quickly removed at the level of the bladder neck. The bladder body was cut open vertically and divided into strips with identical length (1.5 mm × 5 mm). A strip was mounted longitudinally onto a pressure transducer that was connected online to a PowerLab 4/30 Data Acquisition System (LABCHART 5) and to a Dual Core processor Pentium computer for real-time monitoring of physiological force. The strips were equilibrated for at least 1 h in Krebs–Henseleit (Krebs) solution (composition: NaCl 110 mM, KCl 4.8 mM, CaCl_2_ 2.5 mM, MgSO_4_ 1.2 mM, KH_2_PO_4_ 1.2 mM, NaHCO_3_ 25 mM, and dextrose 11 mM) at 37 °C with the continuous bubbling of 95 % O_2_ and 5 % CO_2._ The strips were continuously adjusted to a 0.5 g resting tension.

In this experiment, α,β-me ATP (100 uM) was initially added. Then, contractile tone was measured following the cumulative application of CAP (10 uM), carbachol (10^−8^, 3 × 10^−8^, 10^−7^, 3 × 10^−7^, 10^−6^, 3 × 10^−6^, 10^−5^ M), and KCl (100 mM). After the contractile response of each compound reached plateau, the strips were washed thrice and allowed to equilibrate further for 30 min before the next compounds were added.

### ATP release measurements

The Krebs solution was collected after CAP was added, and the amount of ATP in the samples was determined using the luciferin–luciferase ATP bioluminescence assay kit (Sigma-Aldrich Corporation, USA). To calculate ATP release, the amounts detected in the samples were corrected for total bladder volume and time.

### RT-PCR

RT-PCR was performed as previously described [8]. Total RNA from the bladder tissue was isolated by a TRIZOL reagent and reverse transcribed into cDNA using an RT-PCR kit (Thermo Fisher Scientific, USA) [14, 15]. The synthesized cDNA was amplified via quantitative RT-PCR on an ABI Prism 7500 system using SYBR Green RT-PCR master mix reagent (Thermo Fisher Scientific, USA). Table 1 presents the expected RT-PCR product sizes and primers used in this study. Data were collected and analyzed using complementary computer software. Gene expression was calculated using the 2^−ΔΔCt^ method and normalized to GAPDH expression in each sample [15].

**Table 1:** Primers used for the RT-PCR analysis of TRPV1, P2X3, and GAPDH

cDNA/product sizes	Sequence (5′-3′)	Amplified length (bp)
GAPDH	Forward primers: ggtgaaggtcggtgtgaacg	233
	Reverse primers: ctcgctcctggaagatggtg	
TRPV1	Forward primers: gtttacctcgtccaccctga	106
	Reverse primers: agagagccatcaccatcctg	
P2X3	Forward primers: tctccagcagagacatcagca	163
	Reverse primers: gggagcatcttggtgaactcag	

### Western blot analysis

Western blot analysis was performed as previously described [8]. Tissue was homogenized, and total proteins were extracted using a total protein extraction reagent kit [16]. Protein concentration was measured using Pierce BCA protein assay kit (Thermo Fisher Scientific, USA). Protein samples were separated on SDS-PAGE gels and transferred to PVDF membranes using a transblotting apparatus (Bio-Rad Laboratories, USA). Membranes were blocked with non-fat milk and subsequently incubated with rabbit anti-TRPV1 (1:1000; Abcam plc, UK) or rabbit anti-P2X3 (1:1000; Abcam plc, UK). After processing washes, the membranes were probed with a secondary antibody (1:2000; Millipore Corporation, USA). Protein bands were visualized using ECL Western blot detection reagents (Bio-Rad Laboratories, USA). The intensity of each target protein band was analyzed and expressed relative to β-actin density.

### Data analyses

Data are expressed as means ± standard deviation of mean (SD). For multiple comparisons, repeated-measure ANOVA (Holm–Sidak) was used. Pairwise and non-pairwise comparisons were performed via Student’s *t*-test. Linear regression analyses were also conducted where appropriate, and ANCOVA was used to compare regression slopes and intercepts. These calculation processes were performed using SPSS 13.0 based on the number of individuals. *P* < 0.05 was considered statistically significant.

## Results

Table 2 presents the results of VSOP. The standard curve of VSOP is shown in Fig. 1b, where *r* = 0.9994 and y = 4830.30 x + 1586.07. The KO mice exhibited significant increases in micturition interval and decreases in voiding volume and micturition times. However, neither SQW high dosage nor low dosage treatment significantly changed the voiding behavior of the KO mice.

**Table 2:** Micturition activities of the WT, TRPV1^−/−^, SQW H, and SQW L groups

Group	Number	Micturition volume (μL)	Micturition times (times)	Micturition interval (mins)
WT	10	93.03 ± 18.6	2.47 ± 0.64	104.3 ± 23.7
KO	8	116.1 ± 21.7*	1.92 ± 0.76*	132.2 ± 32.1*
KO + SQW 1170 mg/kg	8	101.27 ± 35.6	2.07 ± 0.47	123.3 ± 32.1
KO + SQW 585 mg/kg	8	103.7 ± 43.6	2.12 ± 0.51	118.4 ± 36.1

* = P < 0.05; ** = P < 0.01, KO group vs. WT group*. # = P* < 0.05; *## = P* < 0.01, treated group vs. KO group; Student’s *t*-test or Mann–Whitney *U*-test if data are not normally distributed

**Fig. 2:**
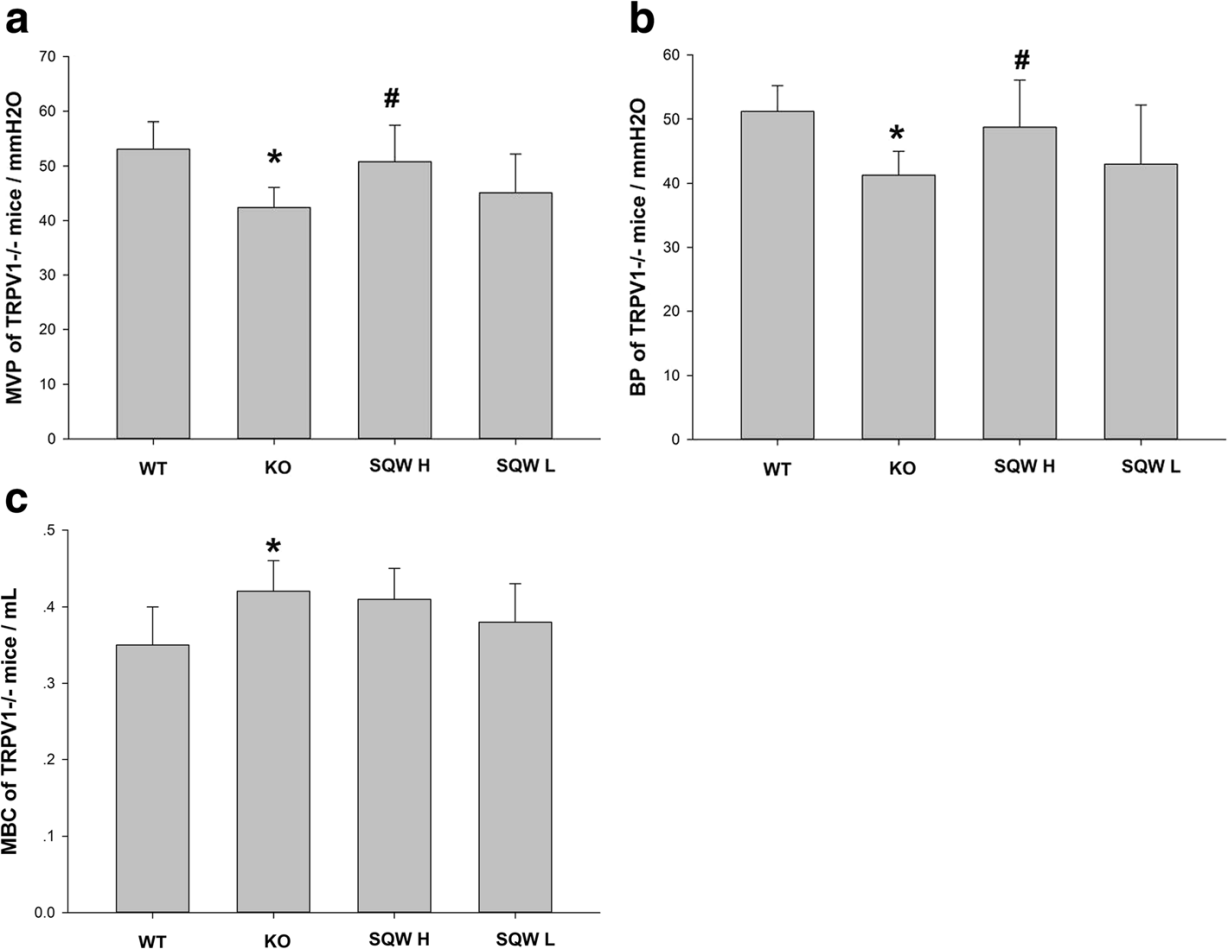
Comparisons of cystometry parameter bladder pressure (BP, MVP), and MBC of the WT, KO, SQW H, and SQW L groups. **a** The Comparisons of MVP of WT, KO, SQW H, and SQW L groups. **b** The Comparisons of BP of WT, KO, SQW H, and SQW L groups. **c** The Comparisons of MBC of WT, KO, SQW H, and SQW L groups. Values are expressed as mean ± SD. * = P < 0.05; ** = *P* < 0.01, KO group vs. WT group; # = *P* < 0.05; ## = *P* < 0.01, treated group vs. KO group. Student’s *t*-test or Mann–Whitney *U*-test if data are not normally distributed

During intravesical instillation with NS, the KO mice exhibited lower bladder pressure (BP, MVP) than the WT mice, and MBC was increased. Compared with the KO mice, the SQW treated mice exhibited a slight increase in bladder pressure (BP, MVP) (Fig. 2a, b), whereas no change was observed in MBC (Fig. 2c).

**Fig. 3:**
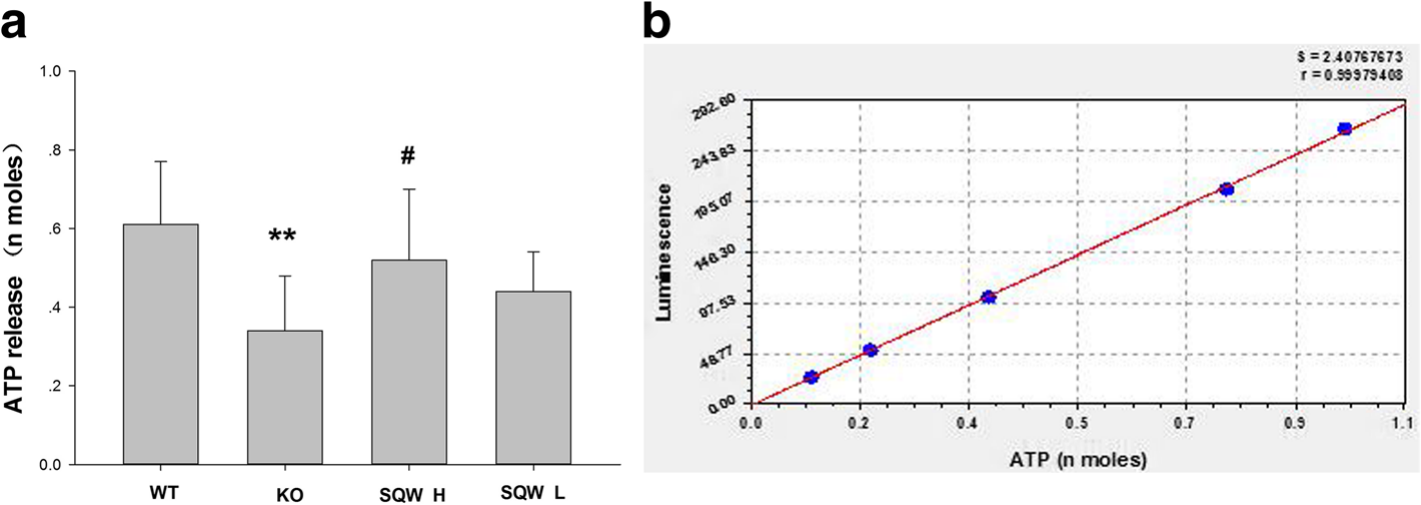
The values of ATP release from the bladder strips of the WT, TRPV1^−/−^, SQW H, and SQW L groups are presented. Values are expressed as mean ± SEM. * = P < 0.05; ** = P < 0.01, KO group vs. WT group*.* Standard curve of the ATP release measurement, where *r* = 0.9998 and y = 2.64 x- 9.48

The standard curve of ATP release is shown in Fig. 3b, where *r* = 0.9998 and y = 2.64 x- 9.48. The ATP release from the bladder strips of the KO mice was significantly lower than that from the WT mice. SQW high dosage treatment slightly increased ATP release; by contrast, no significant difference was found after SQW low dosage treatment (Fig. 3a).

**Fig. 4:**
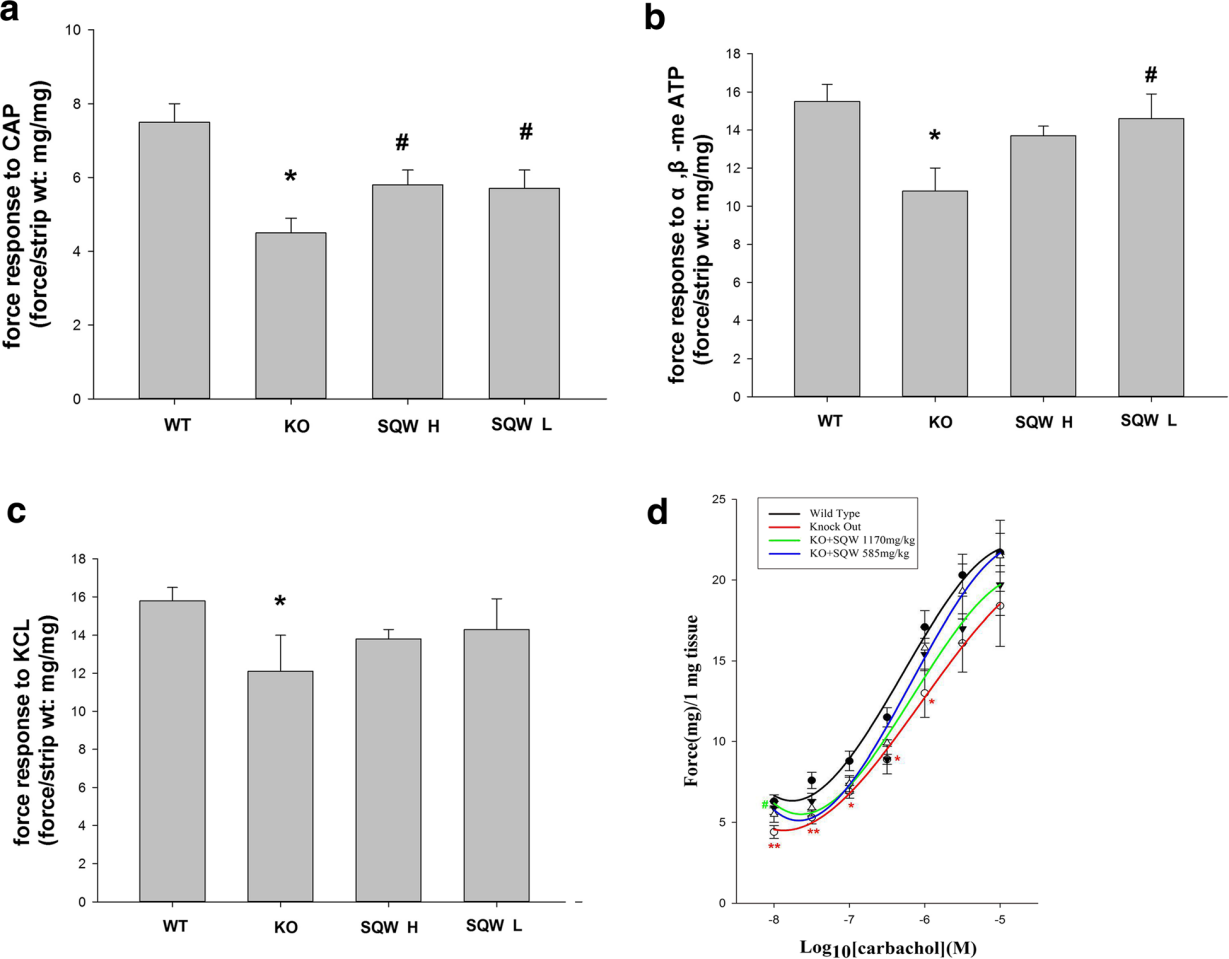
Comparison of bladder strips from different groups in response to α, β-me ATP, CAP, and KCl; and the CRCs of carbachol. **a** Force response to CAP of the WT, KO, SQW H, and SQW L groups. **b** Force response to α,β-me ATP of the WT, KO, SQW H, and SQW L groups. **c** Force response to KCl of the WT, KO, SQW H, and SQW L groups. **d** CRCs of carbachol of the WT, KO, SQW H, and SQW L groups. Values are expressed as mean ± SD. * = P < 0.05; ** = *P* < 0.01, KO group vs. WT group

Force response of TRPV1^−/−^ mice bladder smooth muscle strips to α,β-me ATP (100 uM), KCl (100 mM), and carbachol (10^−8^, 3 × 10^−8^, 10^−7^, 3 × 10^−7^, 10^−6^, 3 × 10^−6^, 10^−5^ M) was significantly reduced (Fig. 4b, c, d), whereas no response to CAP (10 uM) (Fig. 4a), which were observed increase the contraction of the bladder strips of the WT mice.

**Fig. 5:**
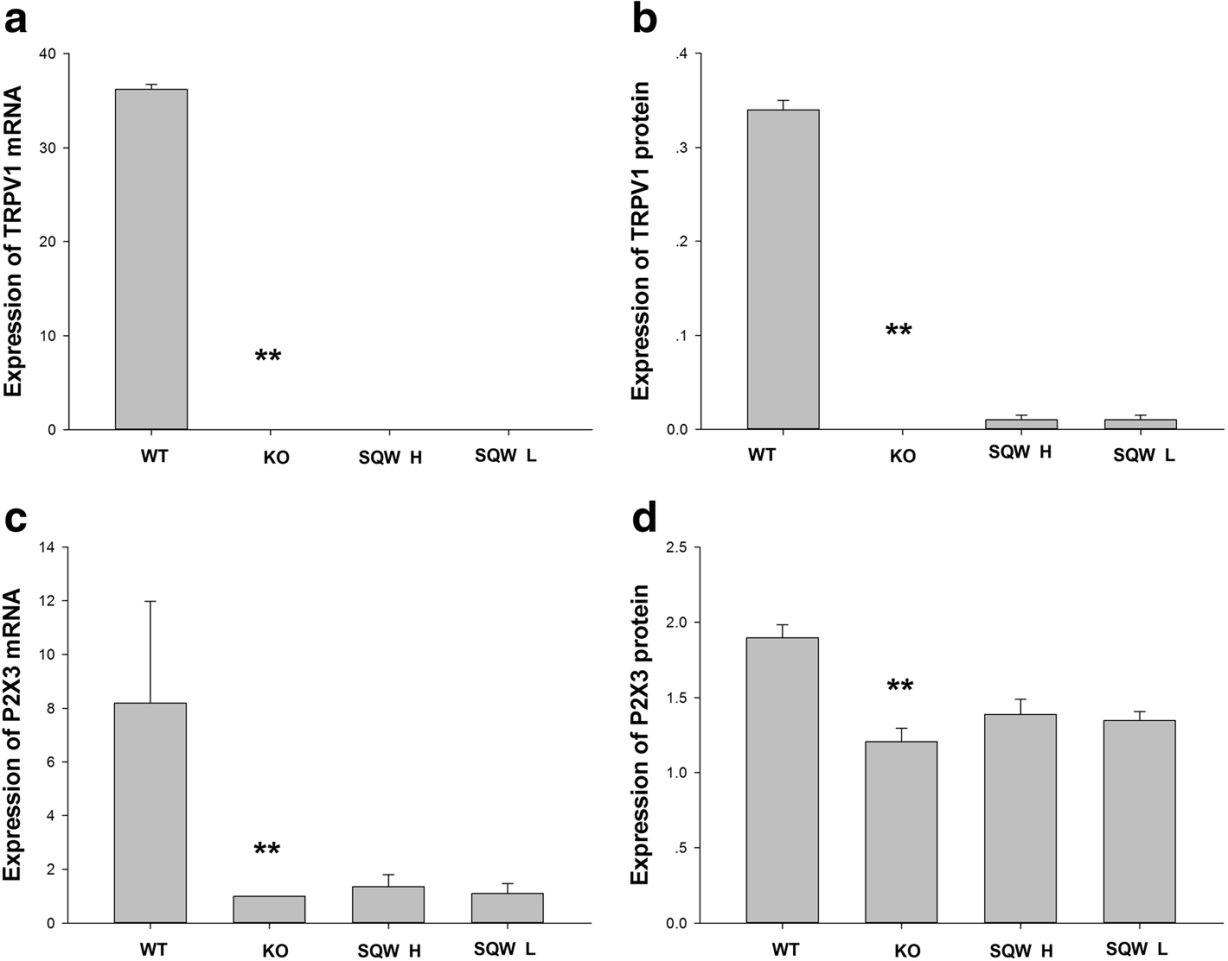
Effects of SQW treatment on TRPV1 and P2X3 protein expression in the bladder. **a** The mRNA expression of TRPV1 in the mice bladder. **b** The protein expression of TRPV1 in the mice bladder. **c** The mRNA expression of P2X3 in the mice bladder. **d** The protein expression of P2X3 in the mice bladder. Values are expressed as mean ± SD. # = *P* < 0.05, ## = *P* < 0.01 vs. OAB model group. For the protein expression, the groups order of immunoblot membranes from left to right are: WT group, SQW H group, SQW L group, KO group

According to the results of the RT-PCR and western blot analysis (Fig. 5a, b, c, d), the TRPV1^−/−^ mice exhibited no TRPV1 expression and a lower expression level of P2X3 in the bladder compared with the WT mice. Similar results were obtained for the SQW H and the SQW L groups.

## Discussion

TRPV1 was observed functions as a chemical and thermal sensor in vivo and plays an essential role in inflammation, nociception, and heat perception by creating a TRPV1 KO mice model [17]. Another research of Birder et al. on mice lacking TRPV1 receptor inhibited increased frequency of urination and increased frequency of low-amplitude contractions in such animals [4]. These observations clearly indicate the involvement of TRPV1 receptors in the micturition process, not only in pathological states, but also in normal conditions. In previously study, the TRPV1 KO mice inhibited attenuation of bladder pressure during intravesical instillation with NS. And the in vitro bath study of the bladder strips of the KO mice showed weakened force response to α, β-meATP, carbachol in gradient concentration, and KCl. But no wonder had no response to CAP. These compounds subsequently elicited smooth muscle strip excitation in the WT mice. Those might drived the TRPV1 KO mice exhibited micturition interval extension, and the decrease of micturition times within a certain period. CAP which located on non-selective ion channels with high permeability for Ca^2+^ ions is ligand of vanilloid receptors. CAP can cause a lower threshold of excitability of these receptors and lead to sensitization and activation [18]. Therefore, no effect was observed in in vitro studies of animals lacking TRPV1 receptors.

Previous study also showed that TRPV1^−/−^ mice exhibited no expression of TRPV1 in both RT-PCR and western blot analysis, along with low ATP content release from bladder strips and low mRNA and protein expression level of P2X3. Stimuli by second messenger, such as ATP can cause increased expression in sensory neurons (capsaicin- sensitive fibers) leading to sensitization of sensory fibers, consequently leads to functional disorders of the lower urinary tract (especially urinary bladder) [19]. Generally, activation with capsaicin can increase intracellular calcium, evoke transmitter (such as ATP) release, and elicit transient currents [4, 20], which consequently activates P2X3 receptors lead to bladder smooth muscle contraction [7]. In contract, mice lacking TRPV1 have no expression of TRPV1 in bladder, inadequate neuron sensitivity lead to Ca^2+^ ions permeability decreased and caused ATP release reduced which related to lower P2X3 expression level. Consequently bring down the contractility of bladder smooth muscle, which can be observed in the in vitro study that KO mice inhibited weakened force response to the compounds supposed to exciting smooth muscle. And eventually caused micturition reflex delay. This result provided proof for the hypothesis on how TRPV1 would affect bladder contraction.

The detailed knowledge of the TRPV1 of lower urinary tract and its interactions appears to be of great clinical significance, as well as being a prerequisite for proper treatment of functional disorders of the urinary tract [21]. In our latest study, we have provided a part of the scientific foundation of SQW is efficacy in recovering bladder function, which is related to the modulation of the TRPV1 expression in bladder [8]. Current study aimed to investigate the mechanisms of SQW on modulating the function of TRPV1 using TRPV1 KO mice. The results indicated that SQW could not improved the voiding behavior of the KO mice. This finding is probably related to the inconspicuous effect on bladder pressure during intravesical instillation. Meanwhile, the phenomenon of urodynamic parameter improvement among OAB rats after treatment with SQW was not observed in mice that lacking the TRPV1 gene. Furthermore, SQW treatment did not significantly affect the force response to α,β-meATP, CAP, carbachol in gradient concentration, and KCl. Although the level of ATP release slightly increased, the expression of P2X3 did not change significantly. According to the aforementioned study, we speculate that SQW modulates the function of TRPV1 and eventually benefits bladder mechanosensitivity and voiding behavior. This phenomenon is relevant with the functional TRPV1 signal and the physiological vanilloid-sensitive afferent neurons transmission.

## Conclusions

The main findings of the present study are as follows. Firstly, reduce ATP release from the bladder and P2X3 secreted from nerves of mice without TRPV1 may result in decrease bladder pressure and voiding reflex delay lead to micturition interval extension. Secondly, the results of SQW intervene on the TRPV1^−/−^ mice exhibited slightly increased ATP release along with insignificant difference on TRPV1 and P2X3 expression and bladder detrusor contractile activities compared with the KO control group. Even though SQW can improve the bladder function in some degree, is still far more than enough to recoverd to normal of TRPV1 KO mice. Meanwhile, the effective SQW is hard to function normally on bladder function of mice without TRPV1. Therefore, we speculate that the treated effect of SQW on bladder function is related to the modulated the TRPV1 signaling which dominated the afferent nerve conduction and the relevant neurotransmitter secretion.

## Additional file

Additional file 1: The quality control of Suoquanwan (SQW). (PDF 139 kb)

## Abbreviations

BOO: Bladder outlet obstruction
BP: Bladder pressure
CAP: Capsaicin
CNS: Central nervous system
KO: Knock out
LUTS: Lower urinary tract symptoms
MBC: Maximum bladder capacity
MVP: Maximum voiding pressure
OAB: Over active bladder
RT-PCR: Real-time polymerase chaim reaction
SQW: Suo Quan Wan
TRPV1: Transient receptor potential vanilloid 1
VSOP: Voiding stain on paper
WB: Western blot
